# Enhancing an Aerospace Grade Benzoxazine Resin by Means of Graphene Nanoplatelets Addition

**DOI:** 10.3390/polym13152544

**Published:** 2021-07-31

**Authors:** Vanessa García-Martínez, Maria R. Gude, Silvia Calvo, Alejandro Ureña

**Affiliations:** 1 FIDAMC, Foundation for the Research, Development and Application of Composite Materials, Avda. Rita Levi-Montalcini 29, Getafe, 28906 Madrid, Spain; maria.r.rodriguez@fidamc.es (M.R.G.); silvia.calvo.external@fidamc.es (S.C.); 2 Department of Applied Mathematics, Materials Science and Engineering and Electronics Technology, Universidad Rey Juan Carlos, Móstoles, 28933 Madrid, Spain; alejandro.urena@urjc.es

**Keywords:** nanocomposites, electrical and thermal conductivity, thermomechanical properties

## Abstract

Two different contents of graphene nanoplatelets (GNPs: 0.5 and 2 wt.%) were introduced into benzoxazine resin. The main objective of this work is to obtain a polymeric nanocomposite with multifunctional properties as high electrical and thermal conductivity, maintaining or improving its mechanical performance. The quality of the dispersion, performed with a three-roll calender, was studied. Afterward, a complete characterization of the nanocomposites was carried out in order to analyse the benefits of neat resin. The main features of the nanocomposites such as the mechanical and thermo-mechanical properties, their electrical and thermal conductivity and the behaviour under hygrothermal aging, were evaluated. Results allowed us to confirm that benzoxazine/GNPs composites exhibited an increase in the tensile strength of polymeric matrix which was accompanied by a rise in elongation at break. The electrical and thermal conductivities exhibited a remarkable increment with the addition of 2 wt.% of GNPs (six orders of magnitude and 49% respectively). Finally, the barrier properties of benzoxazine resin were also favoured with the presence of GNPs because the maximum water absorbed in a hot-water environment decreased from 2.52% to 2.14% when 0.5 wt.% of graphene nanoplatelets was added.

## 1. Introduction

Benzoxazine resins are a class of thermosetting polymers belonging to the family of phenolic resins which present interesting advantages compared to other thermosets. In addition to the typical characteristics of phenolic resins, such as heat resistance and fire-retardant properties, they present low moisture absorption, good mechanical performance and high glass transition temperature, and they can be synthesized from inexpensive raw materials [1,2,3]. Compared to the epoxy resins, benzoxazines exhibit good fire-smoke-toxicity (FST) behavior and improved thermal stability (low coefficient of thermal expansion, near-zero shrinkage upon curing and high degradation temperature) [1,4]. This makes them an alternative as a matrix for carbon fiber reinforced polymers (CFRPs) in those applications in which epoxy resins cannot fulfil the requirements related to these properties. They are especially interesting in the aircraft industry for the production of large composite parts, in which the low heat release upon curing in combination with the low cure shrinkage, the room temperature storing ability and the properties previously mentioned provide convincing advantages. Despite this, the properties of benzoxazine resins need to be improved to be more competitive in the aircraft industry. One of their major drawbacks is the limited curing window [1], which can be improved by blending benzoxazine with epoxy resin. The resulting benzoxazine-based resins present better processability, with a wider process window, and additional benefits that will depend on the polymers formulation and ratio [2].

Similar to the approach taken for the enhancement of the epoxy resins in the last two decades, several research projects have addressed the improvement of specific properties of benzoxazines by adding different types of nanofillers. In particular carbon nanotubes have been used with the aim of improving their mechanical behavior [5,6] or electrical conductivity [7]. More recently, several researchers added graphene oxide to BZ resins. Most of them focus on the synthesis of nanocomposites and their thermal characterization, reporting improvements in the thermal stability (both decomposition temperature and coefficient of thermal expansion) and thermal conductivity [8,9,10]. The oxygen functionalities of graphene oxide provide stronger interfaces between the nanofiller and a thermoset matrix. For this reason, graphene oxide or functionalized graphene are the preferred options for the improvement of mechanical behavior [11]. However, when the priority is the increase in the electrical conductivity, graphene nanoplatelets (GNPs) are more suitable due to the lack of functionalities [12,13,14].

Up to now, only a few papers report the synthesis and characterization of graphene nanoplatelets/benzoxazine nanocomposites. This paper is a continuation of the work that started with the analysis of the influence of graphene nanoplatelets in the cure reaction of a benzoxazine-based resin [15]. It was demonstrated that the GNPs used in the present work have an accelerating effect on this reaction. The present paper goes one step further in the characterization of the resulting nanocomposites. The main aim is the improvement of the electrical and thermal conductivities of the BZ-based matrix while maintaining the mechanical behavior and the glass transition temperature.

## 2. Experimental

### 2.1. Materials

GNPs were purchased from XG-Sciences Inc. with the commercial name xGnP M-15. The average platelets lateral size was 15 µm, the average particle thickness was lower than 6 nm, the typical surface area of 120 to 150 m^2^/g and the carbon content was more than 99%. The matrix employed was an industrial benzoxazine-based resin named Loctite BZ9102 which was provided by Henkel Corporation. This polymeric matrix is a one-component system that has been intended for infusion and injection processes in aeronautical applications. This resin can be stored for up to 6 months at room temperature and over one year in a freezer and it is particularly suitable for the manufacture of large parts due to the broad process window, low exotherm and cure shrinkage.

### 2.2. Methods

#### 2.2.1. Preparation of Nanocomposites

**Figure 1:**
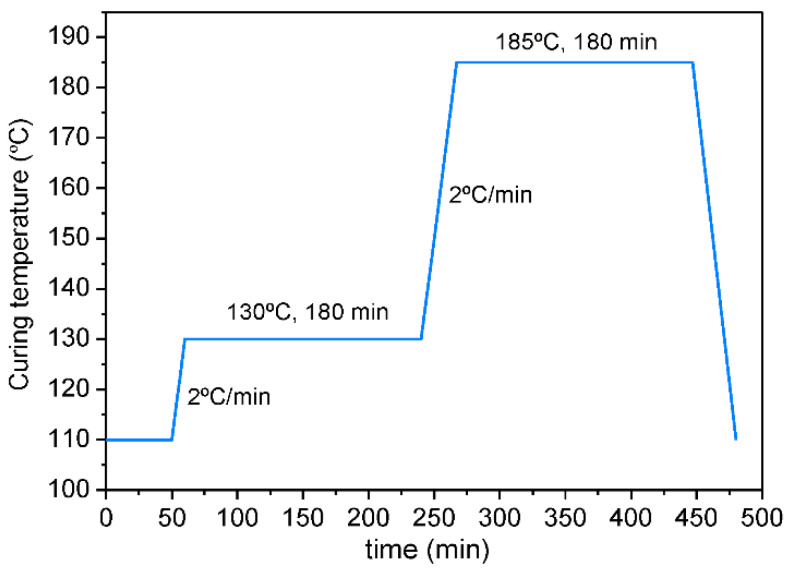
Cure cycle for neat benzoxazine and its nanocomposites.

Graphene/benzoxazine nanocomposites were prepared by means of three-roll calendar (Exakt 80E GmbH) in seven steps optimized by Moriche et al. in previous studies [16]. The gap between each pair of rolls was reduced in every step progressively maintaining a constant rolling speed of 250 rpm. In addition, rolls were water heated at 50 °C in order to reduce the viscosity of the resin. Afterward, dispersions were degassed in a vacuum at 105 °C for 15 min. Two nanocomposites with 0.5 and 2 wt.% of GNPs were prepared. These mixtures were poured into steel molds, previously treated with a mold release agent, and then cured in an oven according to the cure cycle suggested by the resin’s manufacturer (Figure 1). Finally, nanocomposites were demolded and post-cured in an oven at 200 °C for 1 h.

#### 2.2.2. Measurements

Graphene nanoplatelets were characterized by X-ray diffraction (XRD) with an X’Pert PRO diffractometer from Panalytical employing Cu Kα (λ = 1.5406 Å) radiation source operating at a voltage of 45 kV and 300 mA of electric current. This nanoreinforcement was scanned over a range of diffraction angles 2θ from 10° to 80°. Moreover, Raman spectra of GNPs were collected using a Horiba Jobin-Yvon HR800UV spectrometer with a 632.8 nm excitation wavenumber He-Ne laser. Transmission electron microscopy (TEM) images were also taken on a Philips Tecnai 20 T microscope of 200 kV.

The dispersion state of GNPs into benzoxazine was evaluated by means of optical microscopy over uncured samples with Leica DMR optical microscope using the Image-Pro Plus image software. In addition, FEG-SEM analysis was performed in the cryogenic fracture surfaces of nanocomposites which were coated by sputtering with a thin layer (5–10 mm) of Au (Pd). The experimental conditions of the sputtering were 30 mA for 120 s (Baltec, SCD-005 sputter).

A complete characterization of nanocomposites was carried out, including the measurement of the electrical and thermal conductivity, the study of the thermal and mechanical properties as well as their hydrothermal behavior. Electrical conductivity was measured with a four probes method using a Source-Meter Unit, model Keithley 2410 from Keithley Instruments with an interface GPIB to a computer. A voltage sweep was applied to the specimens and the current intensity was measured. The determination of electrical resistance was estimated from the slope of the current–voltage versus intensity curve and then electrical conductivity was calculated taking into account the dimension of the specimens (10 × 10 × 1 mm). Silver paint was applied to both ends to guarantee good contact between the copper electrodes and the specimen. Five specimens per composition were employed to evaluate this property.

To determine the thermal conductivity and thermo-mechanical properties, only three specimens per composition were tested due to the high repeatability of the results. The thermal conductivity was measured at room temperature with a thermal constants analyzer Hot Disk TPS 2500S that employs a transiently heated plane composed of a double spiral electrically conducting pattern which is embedded between two thin films of Kapton. The sensor is placed between two pieces of the sample and an electrical current is applied. As a result, an increase in temperature is detected and directly related to the resistance variation because this sensor can be employed as a heat source and as a dynamic temperature sensor simultaneously.

A Dynamic Mechanical Analyzer (DMA), Q800, from TA Instruments in a single cantilever clamp was employed to evaluate the thermo-mechanical properties. A heating ramp of 5 °C/min from room temperature to 300 °C was applied at 1 Hz frequency. The maximum tanδ curve was employed to determine the α-relaxation identified with the glass transition temperature.

Tensile tests were performed in MTS 370 machine with 100 kN of capacity and Epsilon longitudinal extensometer was employed. All tests were carried following the ASTM D638-2 standard employing five type VI specimens and a speed of 5 mm/min.

Finally, their behavior under hygrothermal ageing was measured by immersion in water at 70 °C in accordance with EN 2378. Before ageing, samples were dried in an oven (Controltecnica, SLW 240 STDFFF) at 70 °C until a constant mass was obtained. Water absorption was studied with gravimetric measurements in a Mettler Toledo, XP205 Delta Range Analytical balance with a precision of 10^−5^ g. At selected times, specimens were removed from the environmental chamber (Dycomental, CCK-70/80), drying superficially with paper and cooling to room temperature before their measurement. A total of two specimens per composition were characterized confirming that measurements presented a 0.2% of coefficient of variation. Water uptake of all samples was determined by the following expression:

(1) $$Water\;uptake,\;M_{t}\;\left(\%\right)=\frac{W_{t}-W_{i}}{W_{i}}\times 100$$

where *M_t_* is the water absorbed at a time-specific immersion period and *W_t_* and *W_i_* are sample weight at respective immersion time and sample initial weight, respectively. The equilibrium condition was reached when the difference between three successive weightings was carried out at a 168 h interval on the absorption properties and in conformity with the formula:

(2) $$\frac{\left|W_{t-2}-W_{t}\right|}{W_{t}}\leq 5\times 10^{-4}$$

## 3. Results and Discussion

### 3.1. Characterization of GPNs

**Figure 2:**
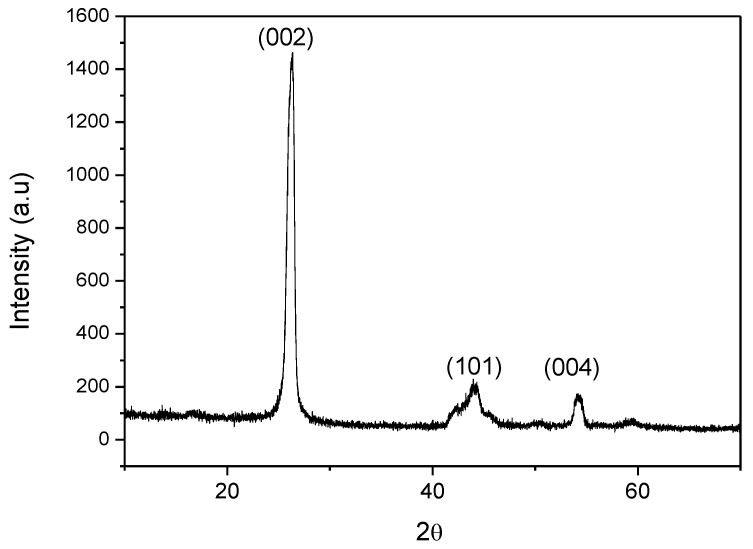
XRD of commercial graphene nanoplatelets.

X-ray Diffraction of commercial graphene nanoplatelets was performed to prove their purity and crystalline structure. The diffraction pattern (Figure 2) showed a sharp peak at 2θ ~ 26.3° corresponding to a d-spacing of 0.339 nm that is related to the graphitic plane (002). The presence of this peak allowed confirming that graphene nanoplatelets have not been completely exfoliated. Scherrer equation [17] was employed to estimate the average domain size from the broadened diffraction peak (002), obtaining a thickness for the GNPs of 12 nm which was double to that indicated by the supplier (lower than 6 nm). In addition, it is worth noting that the thermal and electrical conductive properties of graphene nanoplatelets are closely related to the in-plane crystalline size (L_a_). From the Scherrer expression, this crystalline parameter could also be achieved (30 nm) [18] and this result was considerably lower than the manufacturer datasheet (15 µm) because the crystalline domain is lower than the particle dimension.

**Figure 3:**
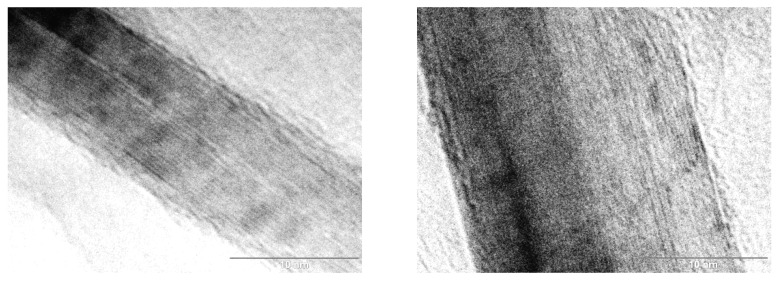
TEM micrographs with the thickness of the GNPs. Scale bar = 10 nm.

TEM analysis was carried out with the aim of verifying the thickness of GNPs (Figure 3) by measuring directly on these micrographs. As it can be appreciated, the particle thickness was statistically in the range of 10–15 nm which is in accordance with the results achieved by XRD.

**Figure 4:**
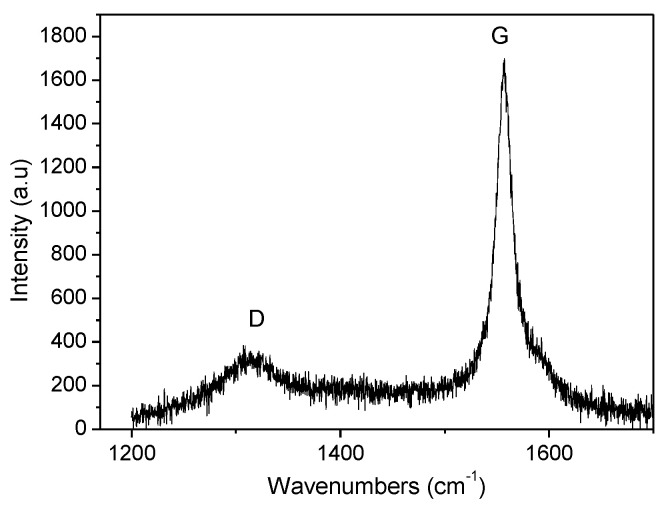
Raman spectrum of graphene nanoplatelets.

Raman spectroscopy is a powerful tool to evaluate defects and residual stresses in graphene nanoplatelets. Figure 4 shows the Raman spectrum of GNPs where two characteristic bands can be appreciated. The pronounced G peak at 1157 cm^−1^ is associated with in-plane vibrations of sp^2^ bonds in both rings and chains. The other dominated band which is known as de D-band was around 1350 cm^−1^ and it is related to the sp^3^ defects of the carbon atoms [16,19]. The relation between the intensity of both bands (I_D_/I_G_) determined the density of defects, so a higher I_D_/I_G_ ratio implies a major density of defects in the structure. Obtained value for these GNPs was 0.15 which confirmed the elevated purity of these GNPs. Moreover, this result was only slightly lower than that acquired by Chiu et al. for the same type of graphene nanoplatelets [20]. From the intensity ratio of the G and D bands the in-plane crystalline size can be also calculated following the relation L_a_ (nm) = 4.4 (I_G_/I_D_) [21,22]. The L_a_ value obtained for GNPs was 29 nm which was very similar to that determined by means of XRD.

### 3.2. Dispersion Degree of GNPs in Benzoxazine Resin

**Figure 5:**
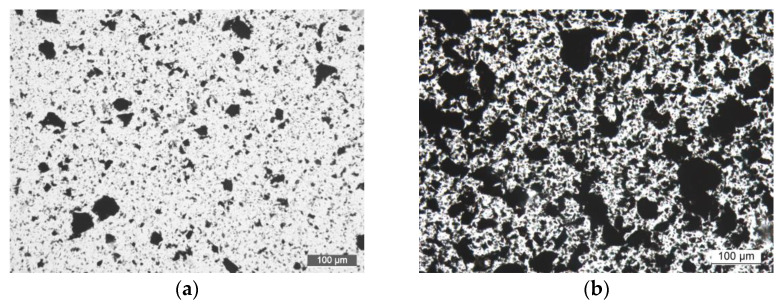

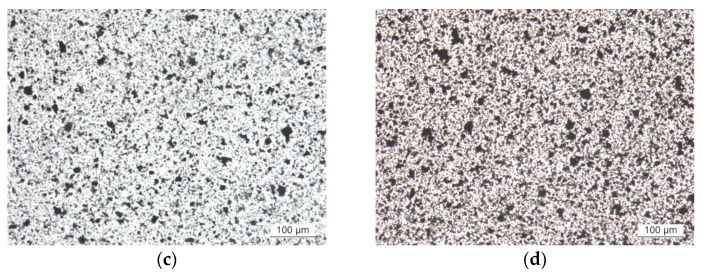
Transmission optical microscopy images of nanocomposites with 0.5 wt.% (**left**) and 2 wt.% (**right**) nanocomposites: before (**a**,**b**) and after (**c**,**d**) the application of three-roll calender at 100×.

It is well known that the selection of a suitable dispersion method is one of the key points in the preparation of graphene/polymer nanocomposites, since the improvement in their final properties highly depends on the quality of the dispersion and the degree of nanoreinforcement exfoliation [23,24]. The major enhancements are achieved when GNPs are completely exfoliated in single layers with the highest aspect ratio. Transmission optical microscopy was firstly employed to characterize the degree of dispersion in the nanocomposites at a micrometer scale. Figure 5 displays micrographs of nanocomposites with 0.5 and 2 wt.% of GNPs before and after the application of the shear forces induced by the three-roll calender. Before applying the dispersion method, both nanocomposites presented dark areas indicating that graphene nanoplatelets are aggregated within the resin. These aggregates are due to strong Van Der Waals forces, large surfaces areas and strong π–π interactions between the graphene layers [25]. As was expected, the presence of aggregates was especially marked in the nanocomposite with the higher amount of GNPs. However, after applying seven calender cycles during which the gap between roll was progressively diminished, the dimension of these aggregates was significantly reduced in both nanocomposites and a homogeneous distribution of these GNPs was achieved. Furthermore, an elevated number of particles with lower dimensions were observed which could be related to the dispersion of the particles initially stacked over others with larger lateral dimensions or with the particles breakage as a result of the calender shear forces.

**Figure 6:**
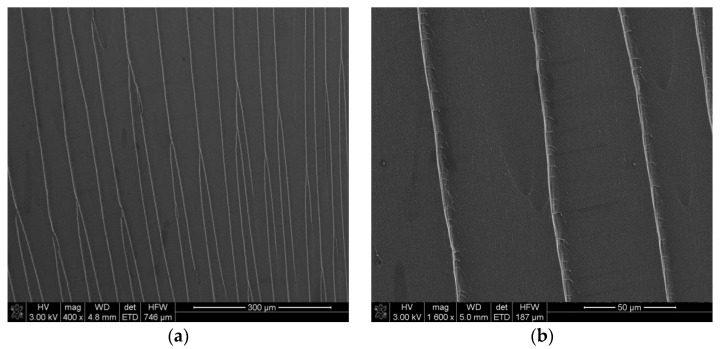

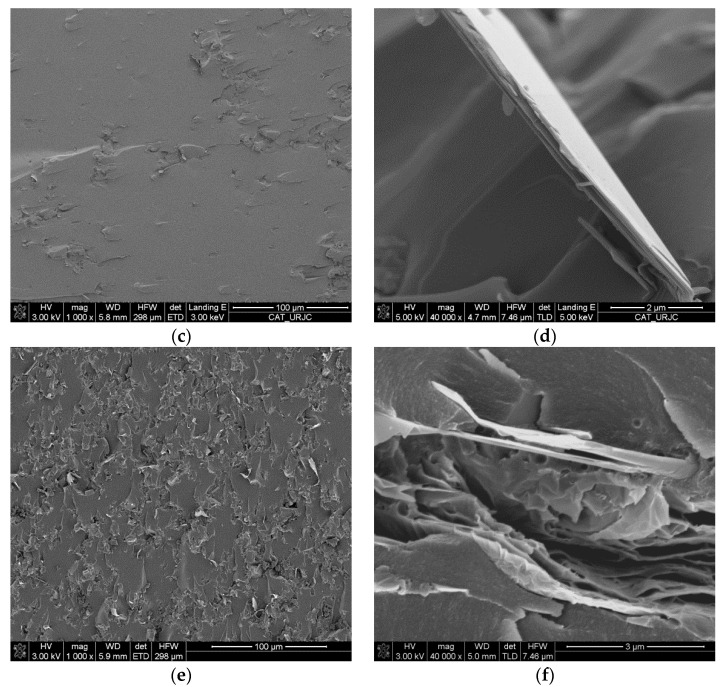
FEG-SEM images of fracture surface: (**a**,**b**) neat benzoxazine, (**c**,**d**) nanocomposites with 0.5 wt.% of GNPs and (**e**,**f**) nanocomposite with a 2 wt.% of GNPs.

To further investigate the performance of the dispersion method, cryogenic fracture surfaces of nanocomposites were evaluated by FEG-SEM (Figure 6). Neat benzoxazine presented a smooth fracture surface (Figure 6a,b) which is characteristic of brittle materials. In contrast, nanocomposites showed a rough fracture surface (Figure 6c,e) which indicated that higher energy was necessary to produce the breakage of these samples. On the other hand, a suitable dispersion can also be appreciated in Figure 6c and e with a uniform distribution and random orientation of GNPs into a benzoxazine resin. Furthermore, these images allowed confirming that GNPs were not completely exfoliated because graphene nanoparticles with a low number of stretched graphitic layers (Figure 6d) and also some thicker aggregates at higher GNPs loadings (Figure 6f) were observed. The employment of three-roll calender produces the exfoliation of external graphene layers, leaving nanoparticles thinner than the initial GNPs [26].

### 3.3. Effect of GNPs Loading in Nanocomposites Mechanical, Physical and Thermal Properties

**Figure 7:**
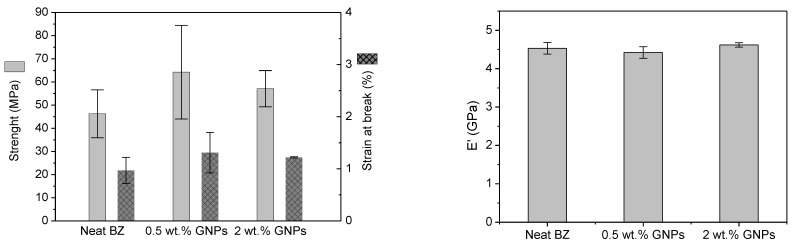
Tensile properties of benzoxazine and its nanocomposites.

The tensile strength and strain at the break of the neat benzoxazine and its nanocomposites are displayed in Figure 7 and Table 1. The neat resin presented a tensile strength and fracture strain of 46.3 MPa and 0.97%, respectively. This property was noticeably enhanced for all nanocomposites but the highest improvement of 40% was obtained when a 0.5 wt.% of GNPs were added to the neat benzoxazine which was accompanied by a rise in the elongation at break. As it was expected, a higher GNPs content into benzoxazine favors their aggregation reducing the strengthening effect of the partial exfoliation phenomenon produced by calendering dispersion. These aggregates may cause a high-stress concentration and premature failure, so this could explain the lower increase in tensile strength (24% vs. 40%) and in the strain at break (26% vs. 35%) for the nanocomposite with a 2 wt.% in comparison with the 0.5 wt.% reinforced one. The tensile ductility increasing was also reported by Alexopoulos et al. for high filler concentrations (>1 wt.% GNPs) [27]. In contrast, the elastic modulus (E’) remained nearly constant when the matrix was modified with graphene nanoplatelets.

**Figure 8:**
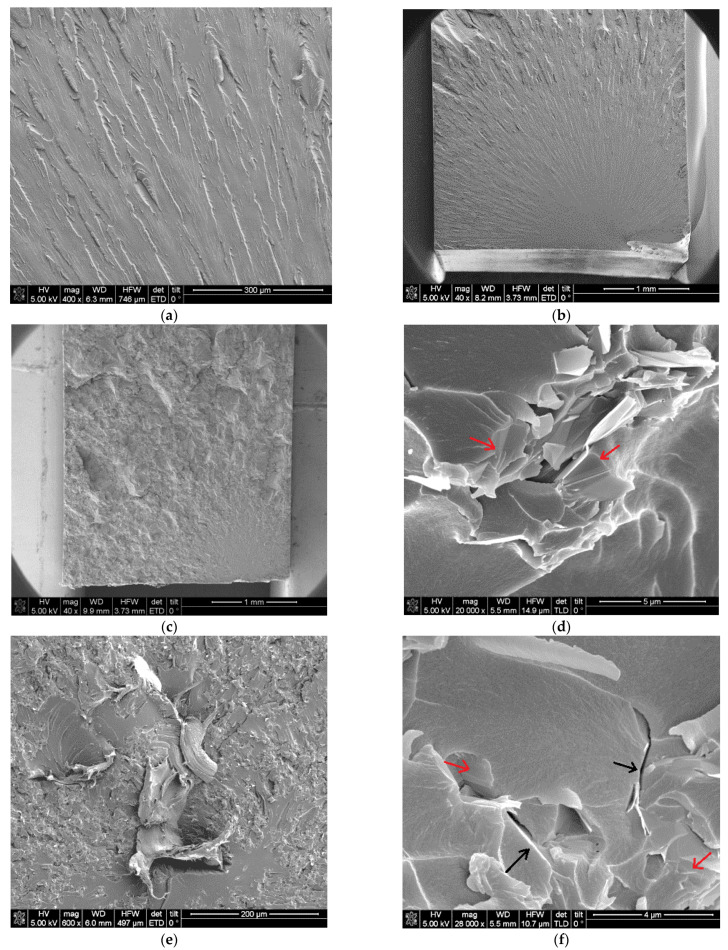
Fracture surfaces: (**a**,**b**) Neat benzoxazine, (**c**,**d**) 0.5 wt.% of GNPs, (**e**,**f**) 2 wt.% of GNPs.

The fracture surfaces morphology was also analyzed by FEG-SEM and characteristic images are displayed in Figure 8. The neat benzoxazine (Figure 8a,b) presented a typical surface of brittle material with a smooth initial failure area and a crack propagation zone which showed “river-like” fracture patterns radially oriented from the crack initiation point. In these typical brittle materials, the crack spread at high velocity in the matrix after its initiation. Polybenzoxazine resins derived from monomeric benzoxazines have usually brittle behaviour due to several types of chain-end groups which lead to termination of the chain propagation by the intramolecular six-membered ring hydrogen bonding [28]. Similar brittle fractures of benzoxazine-type resins have also been observed by other authors [29,30]. When 0.5 wt.% of GNPs was added to neat benzoxazine the appearance of the fracture surface turned visibly different (Figure 8c). The smooth surface of the crack initiation was shorter which was related to a lower fragility and the roughness surface of the propagation area suggested that the crack progress was hindered by the stiff nanoreinforcements. When this surface was observed at higher magnifications (Figure 8d) a good interface between the matrix and graphene nanoplatelets can be appreciated based on the continuity between them. In addition, some single graphene layers bonded to the polymeric matrix (marked with red arrows) and a few nanoparticles with a low number of graphene sheets were also found. It seemed that the weak points which produced stress concentration were the GNPs aggregates whose graphene sheets remained joined by Van der Waals attraction forces, so they tend to slide between them when a load is applied. The initial failure in nanocomposites with 2 wt.% of graphene nanoplatelets was located in the middle of the specimen due to the presence of impurity or heterogeneity which presented a marked plastic behavior (Figure 8e). However, the fracture surface displayed a rougher appearance as a result of the GNPs reinforcement effect which seemed to be uniformly distributed into the matrix. In this nanocomposite, several microcracks which contained small agglomerates of several graphene sheets were also observed as well as some graphene sheets integrated into the benzoxazine (Figure 8f). It appeared that these nanoplatelets did not offer resistance to the crack propagation taking into account the fracture surface plane continuity.

Dynamic mechanical analysis of the nanocomposites was also carried out. Table 2 depicts the storage modulus (E’) values in glassy (at 50 °C) and elastomeric states (at 260 °C) as well as the glass transition temperature associated with the maximum of tanδ curve (T_g_). Storage modulus in the glassy state for benzoxazine with different loading of GNPs remained nearly constant. In contrast, the E’ in the rubbery state slightly increase (9%) when 0.5 wt.% of GNPs was added to the matrix. It is known that a higher storage modulus in the rubbery state is related to a better interaction between the graphene nanoplatelets and polymer chains [31]. There is a relation between the storage modulus in the rubbery state and the molecular weight between two crosslinking points (*M*_x_) which was established by Schlesing et al. [32]:

(3) $$E^{\prime }=\frac{3\mathrm{nRT}}{M_{x}}$$

where n is the density, *M*_x_ the molecular weight between two crosslinking points, R the universal gas constant and T the absolute temperature. The higher value of E’ the lower *M*_x_ which means a more crosslinked polymeric network. The addition of GNPs produced a slight increase in E’_260 °C_, so higher crosslinking was reached in the nanocomposites may be due to the presence of some residual oxygen functional groups in the edges and basal plane of GNPs [15]. The α relaxation temperature experimented a light decrease in nanocomposites regarding neat benzoxazine. The lower T_g_ values with the addition of nanofillers are associated with a weak interfacial adhesion between the polymeric matrix and the graphene sheets [13,33,34], although in this case the glass transition temperature reduction may be related to the presence of some aggregates [35]. Zeng et al. reported a decrease of 7 °C in the T_g_ of a benzoxazine with the incorporation of 0.5 wt.% of graphene oxide (GO) to the polymeric matrix [8]. In the same way, the addition of more than 0.5 wt.% of MWCNTs to benzoxazine resin caused a decrease of glass transition temperature which was more pronounced when the amount of nanofiller into the matrix was higher [36].

**Figure 9:**
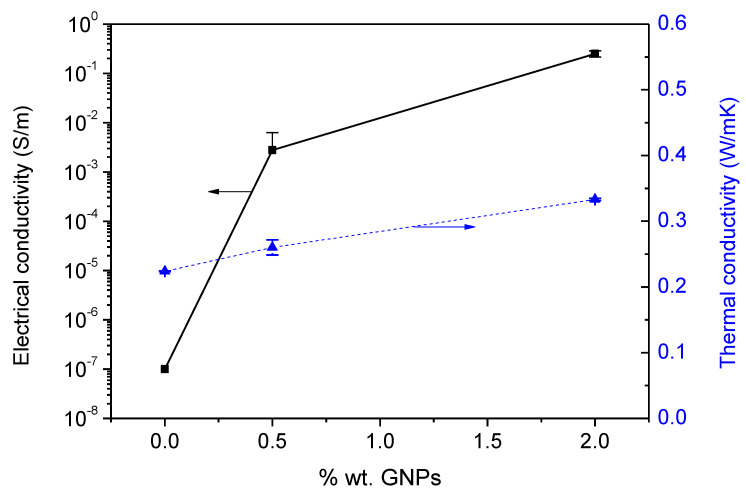
Electrical and thermal conductivity of nanocomposites.

In order to achieve a full characterization of benzoxazine/GNPs nanocomposites, the electrical and thermal conductivity were also measured. The results are displayed in Figure 9. The addition of little GNPs amounts implied noticeable variations in the electrical conductivity. The percolation threshold was close to 0.5 wt.% GNPs because this was the value at which the electrical conductivity raised four orders of magnitude regarding neat resin (~10^−7^ S/m). The reason could be that graphene sheets are in contact with each other as a result of their high aspect ratios; hence the formation of conductive pathways through the nanocomposite could be possible. A new increase in this property until 2.5 × 10^−1^ S/m was achieved when 2 wt.% of GNPs were added to the matrix. This result corroborates that the higher contents of GNPs into the polymeric matrix increase the proportion of partially exfoliated sheets into the polymeric matrix which results in a higher number of physical contacts between individual graphene sheets. This creates a more stable electric percolation network which favors the transport of electric charges through the composite. In this network, nanometric dimensions of graphene sheets also favor the contribution of the tunneling conduction mechanism in absence of electric contact. Apart from this, the intrinsic conductivity of the GNPs also contributes to a marked increase in the effective conductivity of these systems [37]. Although the number of studies related to benzoxazine nanocomposites containing GNPs is scarce in the literature, the electrical conductivity achieved in this work was significantly elevated (at the same filler content) in comparison with others systems, including those with an epoxy matrix. Hence, taking into account the greater electrical conductivity of the benzoxazine nanocomposite with 2 wt.% of GNPs, it could be explored as an alternative lightning strike protection strategy for composite aircraft avoiding the current protection approach based on conductive metal meshes [38]. This solution would allow reducing the weight of composite structures and thus the fuel consumption and environmental impact. Zhang et al. could increase 400 times the conductivity of polybenzoxazine films when 1 wt.% of trichlorophenylsilane modified graphene was added to the polymeric matrix and this rapidly increased conductivity was attributed to the uniform dispersion of the nanofiller throughout the whole film [39]. Results of electrical conductivity reached by Chandrasekaran et al. demonstrated that the application of several calender cycles allows increasing nanocomposite conductivity by several orders of magnitude regarding those prepared with sonication combined with high shear mixing. In addition, the value of electrical conductivity for the nanocomposite with 0.5 wt.% of GNPs (using calender) was very similar to the one obtained in our study (2.8 × 10^−3^ S/m) [40]. When an epoxy resin is reinforced with 0.57 wt.% of thermally reduced graphene oxide and 0.57 wt.% of multi-wall carbon nanotubes (MWCNT), the electrical conductivity of the bi-filler nanocomposites attained to 3 × 10^−3^ S/m which was one order of magnitude lower than the epoxy cured nanocomposite with only 0.57 wt% of MWCNT/(7.8 × 10^−2^ S/m) [41].

On the other hand, the thermal conductivity was also enhanced when GNPs were added to the benzoxazine matrix (Figure 9). The addition of 0.5 wt.% of nanofiller produced an increase up to 0.26 ± 0.01 W/mK while by adding a content of 2 wt.%, the thermal conductivity obtained was 0.33 ± 0.002 W/mK which means an increase of ~16% and ~49% respectively regarding neat benzoxazine. Zhang et al. studied the thermal effect of oxygen functionalities of graphene oxide on thermal properties of reactive benzoxazine nanocomposites and observed that with the addition of 6 wt.% of GO, the thermal conductivity increased ~ 176% regarding pristine benzoxazine [10]. Wang et al. reported values of 0.46 W/mK with the addition of 5 wt.% of GNPs to epoxy matrix [42]. Likewise, an increase of 36% in the thermal conductivity of epoxy resin was reached by Olowojoba et al. when 2 wt.% of thermally reduced graphene oxide was added to epoxy resin [43].

Although the efficient heat propagation in GNPs is mainly related to the diffusion phonons, other contributions as electron propagation, the dispersion state and the shape of the graphene sheet are also significant parameters that participate in the continuous increase in thermal conductivity [44,45,46]. The heat transfer is boosted when a uniform dispersion is obtained and nanofillers present a large lateral dimension and low thickness because the interface between matrix/GNPs is maximized. This interface has a noticeable influence on the thermal conductivity of the nanocomposites. Thus, weak interaction between the GNPs and the polymeric matrix produces an interfacial thermal resistance that hinders the heat flow [21,47]. Moreover, Colonna et al. have confirmed that the improvement of thermal conductivity was closely related to the number of defects in the graphene nanoplatelets. Hence, nanocomposites containing GNPs with a high density of defects in the sp^2^ structure undergo a drop in this property [48].

Finally, all samples were subjected to hydrothermal aging by immersion in water at 70 °C with the aim of evaluating the barrier properties associated with hydrophobic behavior and the planar geometry of GNPs. The water absorption process is a complicated phenomenon influenced by several factors such as the free volume, morphology, hydrophobicity and matrix crosslinking degree among others [49]. When graphene nanoplatelets are added to the polymeric matrix, a strong interface between the matrix and nanofillers restricts the mobility of polymeric chains in the vicinity of high-aspect-ratio GNPs delaying the polymer chain relaxation and reducing the water molecules diffusion [31].

**Figure 10:**
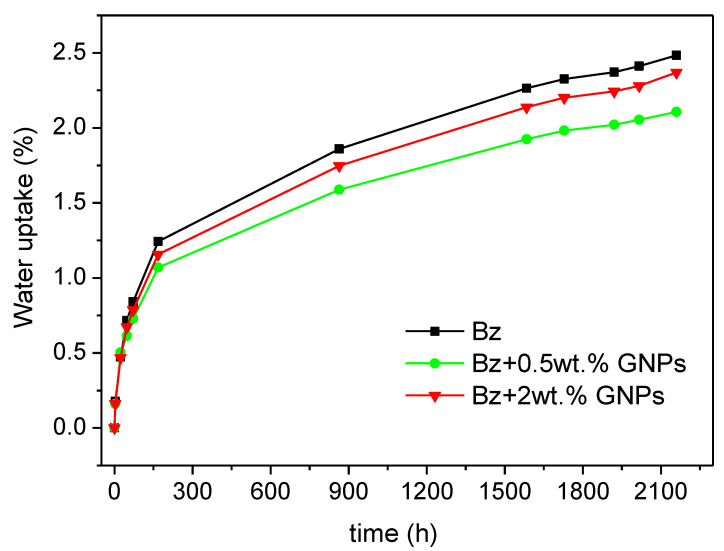
Water uptake for neat benzoxazine and nanocomposites with different GNPs content.

The evolution of water uptake with time is represented in Figure 10. Neat benzoxazine absorbed 2.51% of water at saturation. This value was slightly higher than the one obtained by Ishida and Allen for the benzoxazine based on bisphenol A and aniline (1.9%) [50]. In contrast, when GNPs were added to this matrix the maximum water absorbed decreased from 2.37% and 2.14% for nanocomposites with 2 wt.% and 0.5 wt.% of GNPs, respectively which entails a reduction of 15% (0.5 wt.% Gr) and 6% (2 wt.% GNPs) in the total amount of water uptake. The addition of more GNPs (2 wt.%) did not show much hydrothermal resistance compared with nanocomposite with 0.5 wt.% of nanoreinforcement. It can be explained due to the presence of some aggregates which reduce the specific surface area. In addition, the restriction to intermolecular movement has become ineffective and enhanced water absorption [51].

### 3.4. Summary of Results

The addition of GNPs produced different effects on numerous aspects of the benzoxazine behavior such as their mechanical performance, maximum temperature service as well as thermal and electrical properties (Table 3). The presence of low amounts of GNPs (0.5 wt.%) allowed to obtain interesting advantages in the final properties of the benzoxazine while higher contents of nanoreinforcement were especially beneficial when high electrical and thermal conductivities were required.

## 4. Conclusions

The addition of GNPs to benzoxazine matrix was demonstrated to bring remarkable enhancement in electrical and thermal conductivity properties as well as a slight increase in the mechanical and water uptake behaviour.

A uniform distribution of GNPs and a suitable dispersion into benzoxazine resin was achieved by means of using a three-roll calender. Hence, a complete characterization of these nanocomposites in terms of mechanical, thermal and electrical properties was performed.

A minimum amount, close to 0.5 wt.% of GNPs, was required to achieve the percolation threshold, so conductive pathways through the matrix were created. The electrical conductivity reached 2.5 × 10^−1^ S/m at 2 wt.% of graphene nanoplatelets, a value significantly elevated in comparison with other systems reported in the literature. A noticeable benefit by means of GNPs addition was also observed in the thermal conductivity which increased 49% when the amount of nanofiller in the benzoxazine resin was 2 wt.%. The high electrical and thermal conductivities obtained in this work may allow these nanocomposites to be employed in applications where certain requirements of charge dissipation, electromagnetic shielding, lightning strike protection or anti-ice systems are required. In addition, better barrier properties were achieved when benzoxazine was doped with 0.5 wt.% of GNPs because the maximum water uptake by immersion in hot water decreased 15% regarding the neat thermosetting polymer.

## Figures and Tables

**Table 1:** Results of tensile tests for all samples.

Sample	Strenght (MPa)	E’ (GPa)	Strain at Break (%)
Neat benzoxazine	46.3 ± 10.3	4.5 ± 0.2	0.97 ± 0.25
0.5 wt.% GNPs	64.2 ± 20.3	4.4 ± 0.2	1.31 ± 0.39
2 wt.% GNPs	57.1 ± 7.8	4.6 ± 0.1	1.22 ± 0.01

**Table 2:** Glass transition temperature and glassy/rubbery storage modulus of benzoxazine and its nanocomposites.

Sample	E’_50 °C_ (GPa)	E’_260 °C_ (MPa)	T_g_ (°C)
Neat Bz	4.0 ± 0.2	71.5 ± 1.9	233.6 ± 1.1
0.5 wt.% GNPs	3.9 ± 0.1	77.9 ± 2.2	229.7 ± 0.1
2 wt.% GNPs	4.1 ± 0.1	72.1 ± 3.4	228.0 ± 0.8

**Table 3:** Variation of properties with different GNPs loadings.

% wt. GNPs	Tensile Strength	Tensile Modulus	T_g_	Electrical Conductivity	Thermal Conductivity	Water Uptake
0.5	↑40%	↓ 2.4%	↓ 3.9 °C	↑ 4 orders of magnitude	↑ 16%	↓ 15%
2	↑ 24%	↑ 2.0%	↓ 5.6 °C	↑ 6 orders of magnitude	↑ 49%	↓ 6%
